# Comprehensive Analysis Identifies PI3K/Akt Pathway Alternations as an Immune-Related Prognostic Biomarker in Colon Adenocarcinoma Patients Receiving Immune Checkpoint Inhibitor Treatment

**DOI:** 10.1155/2022/8179799

**Published:** 2022-06-06

**Authors:** Anqi Lin, Tianqi Gu, Xigang Hu, Jian Zhang, Peng Luo

**Affiliations:** Department of Oncology, Zhujiang Hospital, Southern Medical University, Guangzhou, Guangdong, China

## Abstract

**Introduction:**

In recent years, immune checkpoint inhibitors (ICIs) have attracted widespread attention and made breakthroughs in progress towards the treatment of various cancers. However, ICI therapy is selective, and its effects on many patients are not ideal. It is therefore critical to identify prognostic biomarkers of response to ICI therapy. The PI3K/Akt pathway plays important roles in tumor formation and metastasis. However, there are no published reports clarifying the relationship between PI3K/Akt pathway mutations and prognosis for colon adenocarcinoma (COAD) patients receiving immunotherapy.

**Methods:**

We collected data from a COAD cohort from The Cancer Genome Atlas (TCGA) database, including whole-exome sequencing (WES) data, RNA-seq data, and clinical data. We also collected data, including clinical prognosis and targeted sequencing data, from a cohort of COAD patients receiving immunotherapy. We collected 50 COAD patients (Local-COAD) from the Zhujiang Hospital of Southern Medical University and performed targeted sequencing. We analyzed the effects of PI3K/Akt pathway mutations on the patients' clinical prognosis, immunogenicity, and immune microenvironments. Gene set enrichment analysis (GSEA) was used to analyze the significantly upregulated and downregulated signaling pathways. We used these results to hypothesize potential mechanisms by which PI3K/Akt mutations could affect the prognosis of COAD patients.

**Results:**

Univariate and multivariate Cox analyses and Kaplan-Meier (KM) survival curves showed that patients with PI3K-Akt mutations had better overall survival (OS) than those without PI3K-Akt mutations. Genes with significant mutation rates in the two cohorts were screened by panoramic view. CIBERSORT was used to analyze changes in 22 types of immune cells to identify immune activated cells. Similarly, patients in the PI3K/Akt-mutated type (PI3K/Akt-MT) group had significantly increased immunogenicity, including increases in tumor mutation burden (TMB), neoantigen load (NAL), and MANTIS score. Using GSEA, we identified upregulated pathways related to immune response.

**Conclusion:**

PI3K/Akt pathway mutation status can be used as an independent predictor of response to ICI treatment in COAD patients. PI3K/Akt mutations are correlated with improved OS, higher immunogenicity, greater immune response scores, and increases in activated immune cells.

## 1. Introduction

Colorectal cancer (CRC) is the fourth leading cause of cancer deaths worldwide. Approximately 95% of CRC patients suffer from colon adenocarcinoma (COAD), which kills 900,000 people each year. Aging, poor lifestyle, obesity, and smoking have all been shown to increase the risk of COAD [1]. At present, the five-year survival rate of metastatic COAD is only 14% [2]. The treatment methods for COAD include endoscopy, surgical local resection, palliative chemotherapy, and targeted therapy. However, these treatment methods are often ineffective for patients with metastatic COAD [1]. ICIs (immune checkpoint inhibitors) have advanced the treatment of many cancers in recent years [3, 4]. Antibodies that block immune checkpoints can promote the immune system to find and destroy cancer cells, thereby producing an effective antitumor immune response. At present, antibodies against cytotoxic T lymphocyte-associated protein-4 (CTLA-4) and programmed cell death-ligand 1 (PD-L1) are the main ICIs used in clinical practice [5]. However, the overall rates of response to anti-PD-1/PD-L1 and anti-CTLA-4 are low [6], and most patients show primary or acquired resistance to these ICIs [7], which is mainly due to unselective treatment administration [8, 9]. Therefore, it is very important to identify suitable prognostic markers of response to ICIs.

In recent years, studies have shown that mutations in specific pathways and genes can be used as predictive markers of ICI efficacy. NOTCH signaling is associated with tumorigenesis, mutagenesis, and immune tolerance in non-small-cell lung cancer (NSCLC), and mutations in NOTCH can also be used to predict ICI efficacy [10]. Likewise, mutations in *MUC16* are correlated with prognosis in solid tumor patients treated with ICIs [11]. TET signaling mutated-type (TET-MT) patients treated with ICIs also tend to have a better prognosis [12]. Microsatellite instability, tumor mutation burden (TMB), and *POLE/POLD1* mutations are also considered prognostic markers for colorectal cancer patients treated with ICIs [13–15]. However, these prognostic markers are unstable. For example, according to Lichtenstern et al. [2], ICIs are only effective against 30-40% of mismatch repair defects and high microsatellite instability (dMMR-MSI-H) tumors, and dMMR-MSI-H COAD accounts for only a small proportion of all COAD cases.

The PI3K/Akt pathway is activated in various cancers and is considered a promising therapeutic target [16]. PI3K/Akt may promote carcinogenesis by stimulating proliferation, survival, metabolic reprogramming, and invasion/metastasis while inhibiting autophagy and aging. In recent years, some studies have explored the role of the PI3K/Akt pathway in the immune microenvironment. For example, Best et.al [17] found that synergy between the *KEAP1/NRF2* and PI3K/Akt pathways drives changes in the immune microenvironment of patients with non-small-cell lung cancer. O'Donnell et al. [18] also found that PI3K/Akt signaling improves the immune microenvironment of several different types of cancers. These findings suggest that PI3K/Akt pathway mutations may be prognostic markers for COAD patients receiving ICI treatments. Somatic activation mutations in PI3K/Akt are most commonly found in cancer [19], but the effect of PI3K/Akt mutations on patient prognosis remains unclear, especially for colorectal cancer patients receiving ICI treatments.

In the present study, we analyzed the prognosis of COAD patients in an immunotherapy cohort and a TCGA cohort and examined changes in the immune microenvironment and immunogenicity. We determined the effect of PI3K/Akt pathway mutations on the prognosis of colon adenocarcinoma patients receiving immunotherapy and explored potential mechanisms for this effect by analyzing the immune microenvironment and immunogenicity.

## 2. Methods

### 2.1. Clinical Samples and Group Definitions

To study the effect of PI3K/Akt pathway mutations on the prognosis of COAD patients, the TCGAbiolinks package [20] was used to download whole-exome sequencing data, RNA-seq data, and clinical data from the TCGA-COAD cohort. The second cohort was an immunotherapy cohort previously reported by Samstein et al. [15]; patients in this cohort received PD-L1 or CTLA-4 inhibitors. For the immunotherapy cohort, we obtained data on clinical prognosis and targeted sequencing data after immunotherapy. A cohort (Local-COAD) containing 50 COAD patients from the Zhujiang Hospital of Southern Medical University was collected and performed target sequencing. The supplementary method shows details of sample preparation and sequencing. This study has obtained consent from the Ethics Committee of Zhujiang Hospital of Southern Medical University and informed consent of all participating patients. Immunogenic characteristics of Local-COAD are shown in Table S1. We determined the mutation status of each patient by analyzing the number of mutations in the PI3K/Akt pathway. Patients with no PI3K/Akt pathway mutations were defined as PI3K/Akt-wild type (PI3K/Akt-WT), and patients with PI3K/Akt pathway mutations were categorized as PI3K/Akt-mutated type (PI3K/Akt-MT).

### 2.2. Data Acquisition and Preprocessing

TMB can predict patients' response to immunotherapy. According to Chalmers et al.'s definition of TMB [21], we evaluated TMB in the TCGA-COAD and immunotherapy cohorts. Sequencing data for base excision repair (BER), homologous recombination, mismatch repair (MMR), single-stranded breaks (SSB), double-stranded breaks (DSB), nucleotide excision repair (NER), nonhomologous end joining (NHEJ), Fanconi anemia (FA), and their combined gene sets were downloaded from the Molecular Signatures Database (MisgDB) [22], and mutations in these DNA damage repair (DDR) pathways were analyzed. Published studies were used to define neoantigen loads, immune-related genes, and immune scores [23, 24]. We used the CIBERSORT algorithm to evaluate the proportions of 22 types of immune cells in the tumor microenvironment [25]. The input files were the gene expression matrix normalized by FPKM and the database file containing marker genes of 22 types of immune cells. The running code was from the official website of CIBERSORT (https://cibersort.stanford.edu/), and the number of calculations for “permutations for significance analysis” was 1000 times. The R package “limma” [26] was used for the difference analysis, and the package “clusterProfiler” [27] was used for enrichment analysis, result output, and graphic display of Gene Ontology (GO) and Kyoto Encyclopedia of Genes and Genomes (KEGG) terms.

### 2.3. Statistical Methods

All relevant statistical tests were performed in R. For univariate and multivariate Cox regression analyses of the immunotherapy cohort, hazard ratios (HRs) and 95% confidence intervals (95 CIs) were used to evaluate the effect of PI3K/Akt pathway mutations on clinical prognosis. A Kaplan-Meier (KM) curve was used to analyze the relationship between pathway mutations and OS. R packages “survival” and “survminer” were used for survival analysis and visualization [28, 29]. Extraction and analysis of mutation data were achieved through R package “Maftools” [30]. R package “ComplexHeatmap” was used for heat map visualization [31]. Fisher's exact test was used to compare between-group differences in classified variables. Between-group differences in continuous variables were compared using a Mann–Whitney *U* test. The log-rank *P* was used to compare the statistical differences in the KM survival analysis, and *P* < 0.05 was considered statistically significant.

## 3. Results

### 3.1. The Number of PI3K/Akt Pathway Mutations Can Be Used as an Independent Factor to Predict the Prognosis of COAD Patients Receiving Immunotherapy

**Figure 1:**
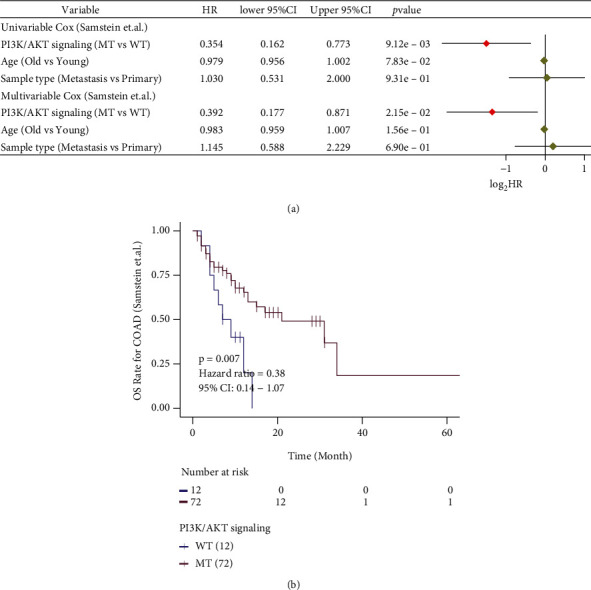
Results of the univariate and multivariate Cox regression analyses and KM survival curve of the immunotherapy cohort. (a) The results of the univariate and multivariate regression analyses are displayed with a forest map. The main part of the forest map is used to show the HR and 95% confidence intervals. The factors associated with good prognosis are HR < 1, and those associated with poor prognosis are HR > 1. (b) The KM curve of the immunotherapy cohort (Samstein et al.) predicted OS (*P* < 0.01, log-rank test).

To explore the effect of PI3K/Akt pathway mutation status on the prognosis of COAD patients receiving immunotherapy, we constructed a univariate Cox model with PI3K/Akt pathway mutation status, age, and sample type as variables. In the immunotherapy cohort, we found that age (elderly vs. young) and sample type (primary vs. metastasis) were not related to the prognosis, while the PI3K/Akt pathway mutation status was closely related to patient prognosis (HR = 0.354, 95% CI: 0.162-0.773, *P* < 0.05; Figure 1(a)). To eliminate potential interference from other factors, a multivariate Cox model was used to analyze other factors influencing patient survival. Results from this analysis showed PI3K/Akt pathway mutation status could be used as an independent predictor of prognosis in the immunotherapy cohort (*P* = 0.021, HR = 0.392, 95% CI: 0177-0.871; Figure 1(a)). According to the KM curve, patients in the PI3K/Akt mutation group had better OS than patients without PI3K/Akt pathway mutations (*P* = 0.007, HR = 0.38, 95% CI: 0.14-1.07; Figure 1(b)).

### 3.2. Mutation Panorama and the Relationship between PI3K/Akt Pathway Mutations and Clinical Features

**Figure 2:**
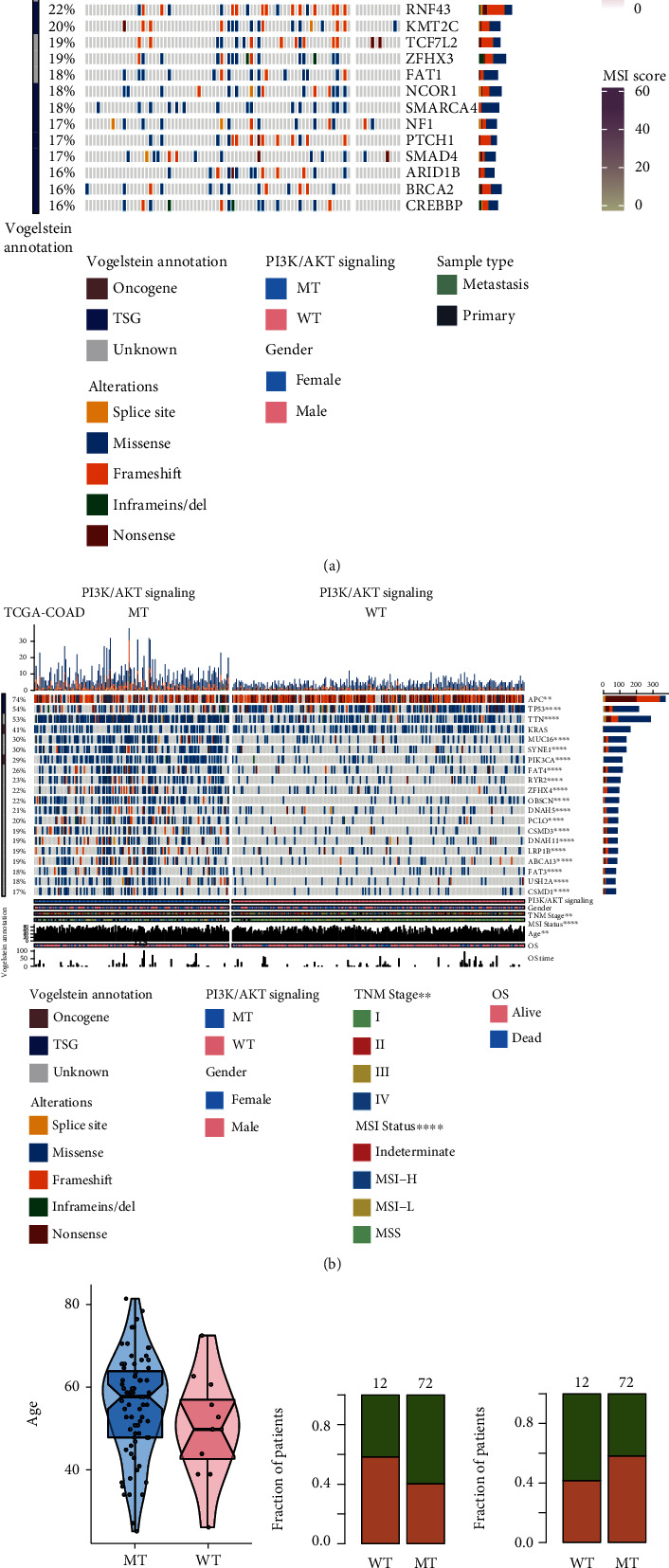
Gene mutation panorama of colorectal cancer patients in the immunotherapy cohort (a) and TCGA-COAD cohort (b) and the relationship between PI3K/Akt pathway mutations and clinical characteristics (c). (a) The 20 genes with the highest mutation frequencies in COAD patients in the immunotherapy cohort and their corresponding clinical characteristics are displayed. The mutation frequencies of PIK3CA, ARID1A, and PTPRS were significantly increased in the PI3K/Akt-MT group. Yellow represents cleavage site mutations, blue represents missense mutations, orange represents frame shift mutations, green represents insertion/deletion mutations, and brown represents nonsense mutations. (b) The 20 genes with the highest mutation frequencies in patients in the TCGA-COAD cohort and their corresponding clinical characteristics are displayed. With the exception of KRAS, the mutation frequencies of all other genes changed significantly. Yellow represents cleavage site mutations, blue represents missense mutations, orange represents frame shift mutations, green represents insertion/deletion mutations, and brown represents nonsense mutations. (c) Relationship between PI3K/Akt pathway mutations and clinical characteristics such as age, sex, and sample type differences in the immunotherapy cohort (^∗^*P* < 0.05; ^∗∗^*P* < 0.01; ^∗∗∗^*P* < 0.001; ^∗∗∗∗^*P* < 0.0001; Fisher's exact test).

**Figure 3:**
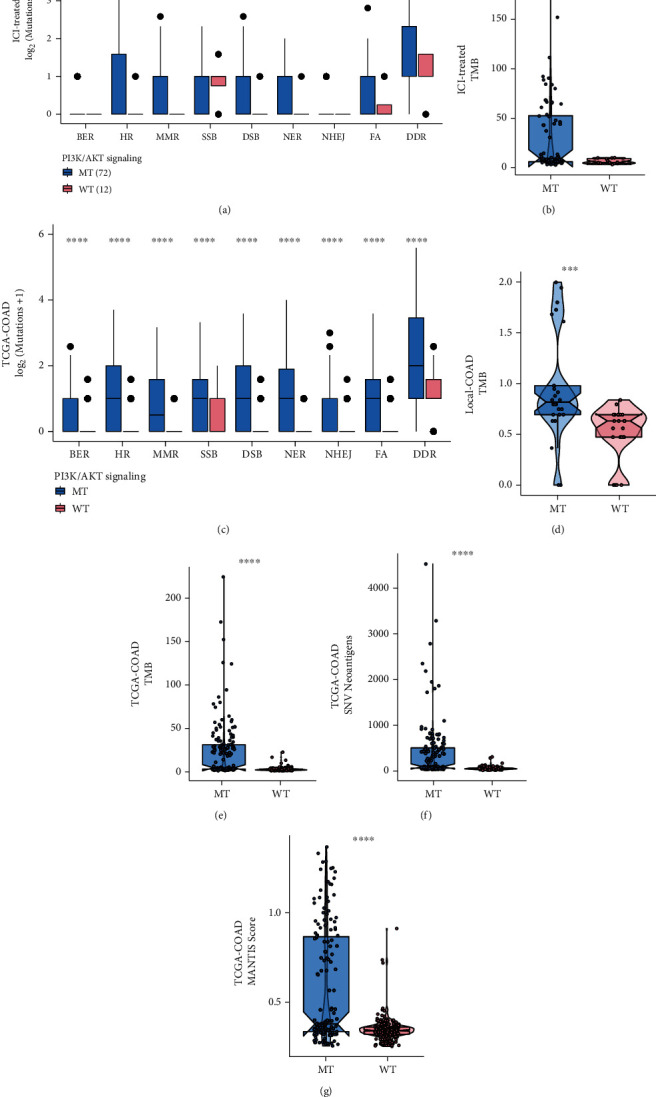
The relationship between PI3K/Akt pathway mutations and enhanced immunogenicity. (a) Comparison of DDR mutations in the PI3K/Akt-MT and PI3K/Akt-WT groups in the immunotherapy cohort. (b) Comparison of TMB in the PI3K/Akt-MT and PI3K/Akt-WT groups in the immunotherapy cohort. (c) Comparison of DDR mutations in the PI3K/Akt-MT and PI3K/Akt-WT groups in the TCGA cohort. (d) Comparison of TMB in the PI3K/Akt-MT and PI3K/Akt-WT groups in the Local-COAD cohort. (e) Comparison of TMB between PI3K/Akt-MT and PI3K/Akt-WT groups in the TCGA cohort. (f) Comparison of NAL between PI3K/Akt-MT and PI3K/Akt-WT groups in the TCGA cohort. (g) Comparison of MANTIS scores of the PI3K/Akt-MT and PI3K/Akt-WT groups in the TCGA cohort (^∗^*P* < 0.05; ^∗∗^*P* < 0.01; ^∗∗∗^*P* < 0.001; ^∗∗∗∗^*P* < 0.0001; Mann–Whitney *U* test).

To explore the effects of PI3K/Akt pathway mutation status in COAD patients at the DNA and RNA levels, we analyzed the top 20 most frequently mutated genes in the TCGA-COAD and immunotherapy cohorts. The clinical characteristics analyzed included PI3K/Akt mutation status, gender, sample type, and MSI score. In the TCGA-COAD cohort, Fisher's exact test showed that except for *KRAS* (*P* = 0.53), the mutation frequency of other genes in the PI3K/Akt-MT group changed significantly compared to the PI3K/Akt-WT group (*P* < 0.05; Figure 2(a)). In the immunotherapy cohort, only *PIK3CA* (38.9% vs. 0%), *ARID1A* (34.7% vs. 0%), and *PTPRS* (30.6% vs. 0%) had higher mutation frequencies in the PI3K/Akt-MT group, while the mutation frequencies of other genes did not change significantly. There were no significant differences in the clinical features such as age, sex, and sample type between the PI3K/Akt-WT and PI3K/Akt-MT groups (*P* > 0.05; Figure 3(c)). The genes driving mutations were usually mutually exclusive. We analyzed the mutual exclusion of the top 20 most frequently mutated genes in the immunotherapy and TCGA-COAD cohorts, and the results are shown in Figure S1.

### 3.3. Effects of PI3K/Akt Pathway Mutations on Immunogenicity

Immunogenicity refers to the ability to induce a humoral or cell-mediated immune response. Differences in immunogenicity can occur due to differences in TMB, DDR, NAL, or MANTIS score [32, 33], and changes in immunogenicity can be determined by comparing changes in these indexes. Immunogenicity can significantly affect the prognosis of patients receiving ICI treatments. In the TCGA-COAD cohort, patients in the PI3K/Akt-MT group had a significantly increased number of mutations in eight DDR and combined pathways (*P* < 0.05; Figure 3(c)). Similarly, patients in the PI3K/Akt-MT group had significantly increased TMB, MANTIS scores, and NAL compared to patients in the PI3K/Akt-WT group (all *P* < 0.05; Figures 3(e)–3(g)), which indicates that mutations in the PI3K/Akt pathway are associated with higher immunogenicity. In the immunotherapy cohort, patients in the PI3K/Akt-MT group had significantly increased mutations in the HR and MMR pathways (*P* < 0.05; Figure 3(a)), while there was no significant difference in the number of mutations in other DDR-related pathways. In both ICI-treated and Local-COAD cohorts, the PI3K/Akt-MT group had significantly higher TMB compared with the WT group (all *P* < 0.05; Figures 3(b) and 3(d)). Importantly, a higher TMB indicates that more tumor neoantigens can be recognized by the immune system, thereby increasing the likelihood of an effective antitumor immune response.

### 3.4. Effect of PI3K/Akt Mutation Status on the Immune Microenvironment

**Figure 4:**
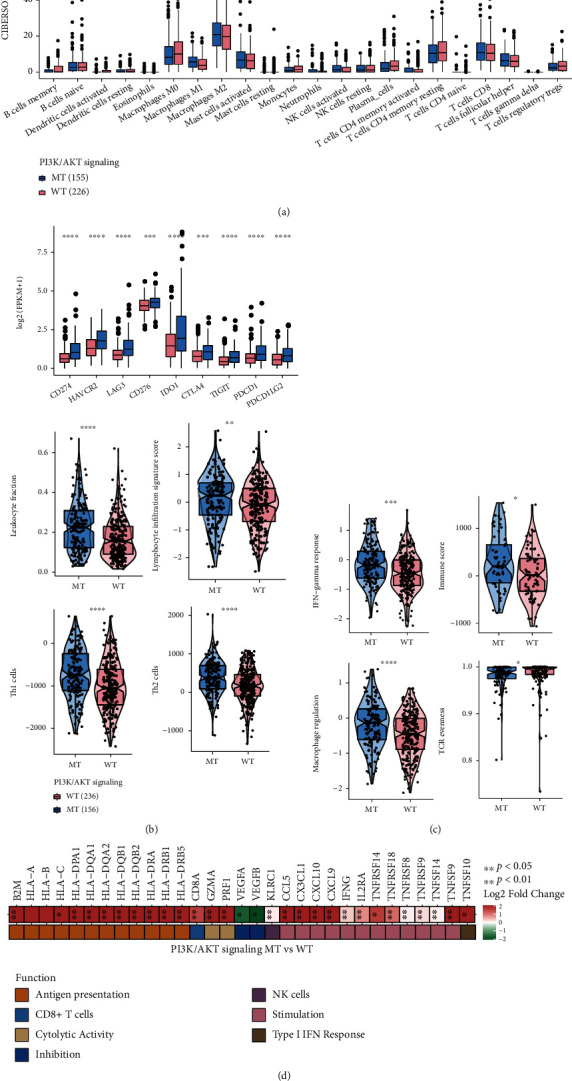
The relationship between PI3K/Akt mutations and immune microenvironment. (a) CIBERSORT comparison of the content of 22 types of immune cells in the tumor microenvironments of PI3K/Akt-MT and PI3K/Akt-WT patients (algorithm running the lm22 signature and 1,000 permissions). (b) Difference in immune checkpoint gene expression between the PI3K/Akt-WT and PI3K/Akt-MT groups. (c) Difference in immune fractions between the PI3K/Akt-WT and PI3K/Akt-MT groups. (d) The functions of immune module genes involved in the PI3K/Akt pathway, including antigen presentation, CD8+ T cells, cytologic activity, inhibition, NK cells, stimulation, and type I IFN (^∗^*P* < 0.05; ^∗∗^*P* < 0.01; ^∗∗∗^*P* < 0.001; ^∗∗∗∗^*P* < 0.0001; Mann–Whitney *U* test).

The tumor immune microenvironment plays a key role in response to ICI treatment. We therefore evaluated the tumor immune microenvironments of COAD patients. In the TCGA-COAD cohort, CIBERSORT analysis identified several types of immune cells that were significantly increased in the PI3K/Akt-MT group (*P* < 0.05; Figure 4(a)), including M1 macrophages, neutrophils, and natural killer (NK) cells, all of which are associated with immune activation and enhanced immune response. By calculating the enrichment score of the immune gene set, we found that patients in the PI3K/Akt-MT group had more sensitive immune responses and increased immune activity than patients in the PI3K/Akt-WT group. Compared to the PI3K/Akt-WT group, immune score indicators such as IFN-gamma production, leukocyte fractions, lymphocyte infiltration signature score, macrophage regulation, T cell receptor (TCR) evenness, and Th1 and Th2 cell infiltration were significantly increased in the PI3K/Akt-MT group (*P* < 0.05; Figure 4(c)).

**Figure 5:**
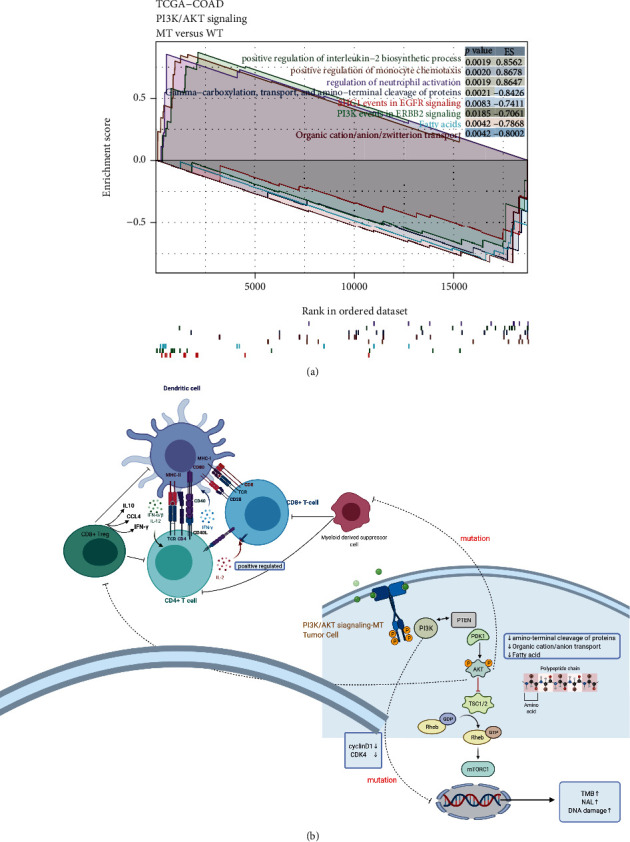
(a) GSEA comparison of upregulated and downregulated genes in the PI3K/Akt-MT and PI3K/Akt-WT groups. The enrichment score greater than 0 indicates that the pathway is upregulated in the PI3K/Akt-MT group, and the enrichment score less than 0 indicates that the pathway is downregulated in the PI3K/Akt-MT group. The color of the pathway name is consistent with the color of the broken line in the figure; *P* value less than 0.05 is statistically significant. (b) The potential mechanism of PI3K/Akt-MT patients with colorectal cancer receiving ICI had a better prognosis.

Immune checkpoint genes are the targets of ICIs. We selected nine types of immune checkpoint genes and analyzed their expression in the PI3K/Akt-MT and PI3K/Akt-WT groups. We found that the expression of nine immune checkpoint genes (*CD274*, *HAVCR2*, *LAG3*, *CD276*, *IDO1*, *CTLA4*, *TIGIT*, *PDCD1*, and *PDC1LG2*) was significantly upregulated in the PI3K/Akt-MT group (*P* < 0.05, Figure 4(b)). We defined genes with similar immune functions as a series of immune modules and analyzed the relationship between these modules and PI3K/Akt pathway mutations. We found that the expression of genes from some immune modules, including antigen presentation, cytolysis, and stimulating immune responses, was significantly increased in the PI3K/Akt-MT group (*P* < 0.05; Figure 4(d)). To thoroughly understand changes in immune function and metabolic pathways, we used GSEA to analyze enriched pathways in the PI3K/Akt-MT and PI3K/Akt-WT groups and to calculate and visualize enrichment fractions. GSEA revealed that some immune pathways, including complement receptor-mediated signaling, monocyte chemotaxis, neutrophil activation, and regulation of interleukin-2 biosynthetic processes, were activated in the PI3K/Akt-MT group, whereas PI3K/Akt pathways such as PI3K events in ERBB2 signaling and SHC1 events in EGFR signaling were inhibited. Pathways related to the synthesis of substances such as fatty acids, organic cation/anion/zwitterion transport, gamma-carboxylation, and amino-terminal cleavage of proteins were downregulated in the PI3K/Akt-MT group (Figure 5(a)).

## 4. Discussion

In the present study, we used TCGA-COAD, Local-COAD, and an immunotherapy cohort to explore the effect of PI3K/Akt pathway mutations on the prognosis of colon adenocarcinoma patients receiving ICI treatments. Through univariate and multivariate Cox regression analyses and KM curves, we found that mutations in the PI3K/Akt pathway can significantly improve the OS of patients receiving ICI treatments. Along these lines, the PI3K/Akt-MT group had significantly increased TMB, NAL, MANTIS scores, and DDR pathway mutations compared to the PI3K/Akt-WT group. Increased PI3K/Akt pathway mutations resulted in an immune microenvironment that was more favorable to inhibit the growth of tumor cells. Meanwhile, GSEA revealed that some immune response pathways were activated in the PI3K/Akt-MT group, whereas some substance synthesis and transport pathways were inhibited in the PI3K/Akt-WT group.

Mutations in the PI3K/Akt pathway were often accompanied by genomic instability. We identified several mutated genes, such as *PIK3CA*, *ARID1A*, and *PTPRS*, whose mutation rate was significantly higher in the PI3K/Akt-MT group of the immunotherapy cohort. Previous studies have shown that about 15%-20% of patients with colorectal cancer carry *PIK3CA* activation mutations, and patients with *PI3KCA* mutations have better OS and progression-free survival (PFS) [34]. However, for colorectal cancer patients receiving ICI treatments, the effect of *PIK3CA* mutations on prognosis is unclear. The present study found that patients in the PI3K/Akt-MT group of the immunotherapy cohort had a higher frequency of mutations in the *PIK3CA* gene, which suggests that *PIK3CA* mutations may improve the prognosis of colorectal cancer patients receiving ICI therapy. In colorectal cancer patients, mutations in *ARID1A* are associated with higher MSI, TMB, and cytotoxic T cell infiltration, which result in improved prognosis [35]. Receptor-type protein tyrosine phosphatases (PTPRs) are a family of transmembrane immunoglobulins. The PTPR family plays an important role in tumorigenesis and regulation of the immune microenvironment [36]. Sun et al. found mutations in *PTPR* family genes lead to better prognosis in NSCLC patients receiving immunotherapy [37], suggesting that this may be a potential mechanism by which PI3K/Akt-MT patients have better immunotherapy efficacy. Thus, genomic instability leads to mutations in genes associated with improved prognosis, and these mutations may explain the better prognosis of PI3K/Akt-MT patients compared to PI3K/Akt-WT patients.

The higher immunogenicity of PI3K/Akt-MT tumors may explain the improved prognosis of these patients compared to patients with PI3K/Akt-WT tumors. We found that PI3K/Akt-MT patients usually had significantly increased TMB compared to PI3K/Akt-WT patients. The TMB usually included coding and somatic mutations, but in some cases, it also included insertions and deletions [38]. A high frequency of somatic mutations can produce new antigens, which increases the probability that tumors are recognized by the immune system. Previous studies have shown that an increased TMB can improve patient responses to PD-L1 inhibitors [39]. Samstein et al. [15] found that COAD patients with high TMB receiving ICI treatments had a better prognosis than patients with low TMB. Further studies have shown that NAL is a better predictor of response to immunotherapy than TMB [40, 41]. NAL can enhance the immune system's ability to recognize tumors by improving T cell immunity and increasing neoantigen-specific T cell content. Schumacher and Schreiber believe that ICIs may partly function through neoantigen-specific T cells [42]. Therefore, NAL can be used as a prognostic marker for cancer patients receiving ICI therapies. In the present study, patients in the PI3K/Akt-MT group had significantly increased NAL, which could explain their better prognosis. DDR pathway mutations are another potential explanation for the improved prognosis of PI3K/Akt-MT patients. In the present study, any mutations in genes in the DDR pathway were defined as DDR pathway mutations. An abnormal DNA damage repair system can lead to DNA damage repair defects, which cause genomic instability and increased TMB. Previous studies have shown that patients with comutations in the DDR pathway have significantly longer OS than those without comutations [43], and of the eight DDR pathways, comutations in the HR-BER and HR-MMR pathways are associated with higher TMB and NAL than other comutation groups. In our study, the PI3K/Akt-MT group had a significantly increased number of HR and MMR comutations, which led to increases in TMB and NAL. To summarize, PI3K/Akt-MT patients had higher TMBs and NALs and an increased frequency of DDR pathway comutations, which led to higher immunogenicity and improved prognosis.

Changes in the immune microenvironment are another potential mechanism for the improved prognosis of PI3K/Akt-MT patients. We found that PI3K/Akt-MT patients had increased infiltration of immune cells such as M1 macrophages and NK cells. NK cells can directly kill tumor cells by cytotoxicity and can also exert immune functions indirectly by secretion of cytokines such as IFN-*γ*, TNF-*α*, CCL3, and CCL4 [44]. However, the tumor microenvironment can interfere with NK cell activation by inducing immune cell dysfunction and inhibiting NK cell antitumor immunity, which can result in tumor immune escape. Under normal circumstances, M1 macrophages can promote antigen presentation and activate Th1 immune responses by overexpression of IL-12 and IL-23, thus killing tumor cells. On the contrary, M2 macrophages can promote tumor angiogenesis, shield immune surveillance, and promote tumor growth and metastasis. In the inhibitory tumor immune microenvironment, the tumor-promoting effects of M2 macrophages are enhanced and the antitumor effects of M1 macrophages are inhibited, which further affects NK cell infiltration. In the present study, CIBERSORT results showed that the abundance of NK cells and M1 macrophages was significantly increased in the PI3K/Akt-MT group. Dendritic cells (DCs) also appeared to affect the activity of NK cells, although the mechanism for this was unclear. One explanation is that NK cells can selectively destroy nonimmunogenic DCs to screen for immunogenic DCs, and this could be the reason that the PI3K/Akt-MT group had decreased abundance of DCs.

IFN-*γ* is a multifunctional molecule with antiproliferation, antiapoptosis, and antitumor functions [45]. IFN-*γ* can upregulate the major histocompatibility complex (MHC) as well as MHC class II transactivator (CIITA), resulting in MHC class II expression [46, 47]. The production of IFN-*γ* is mainly limited to T lymphocytes and NK cells, which can eventually induce an immune response by CD4+ T cells and CD8+ T cells [48]. IFN-*γ* may exert antitumor effects by regulating the cell cycle, inducing tumor cell apoptosis, inhibiting tumor metastasis, and regulating tumor angiogenesis. However, the effects of IFN-*γ* are not ideal in an immunosuppressive microenvironment [49]. In the present study, IFN-*γ* was upregulated in the PI3K-Akt-MT group, which indicated that the immune effects of IFN-*γ* could be restored when the immune microenvironment was improved. It is worth noting that previous studies have found that inhibition of PI3K could downregulate PD-L1 expression and enhance the antitumor effects of IFN-*γ*, and it has been speculated that blockage of PI3K could maximize the antitumor effects of IFN-*γ* [50]. Our research appears to confirm this hypothesis. In conclusion, we found that PI3K/Akt pathway mutations can increase immune cell infiltration and improve the immune microenvironment by upregulation of proinflammatory cytokines.

Through GSEA, we screened for significantly upregulated and downregulated pathways and used this information to speculate the mechanism by which PI3K/Akt pathway mutations lead to a better prognosis (Figure 5(b)). The upregulation of interleukin-2 (IL-2) synthesis leads to increased IL-2 content, and IL-2 enhances the immune function of CD4+ T cells through autocrine regulation [51]. Pathways related to monocyte chemotaxis and neutrophil activation, which further enhance immune function, were also upregulated in PI3K/Akt-MT patients. Signaling pathways related to amino acid modifications, substance transport, and fatty acid synthesis were downregulated in the PI3K/Akt-MT group, thereby inhibiting tumor metabolism. Bone marrow-derived suppressor cells (MDSCs) can expand in cancer hosts and accelerate tumor progression. Previous studies have shown inhibiting AKT and ERK signaling with selective small molecule inhibitors or shRNAs can selectively hinder the differentiation and viability of MDSCs [52]. Therefore, Akt mutations can limit the differentiation of MDSCs and weaken the inhibitory effect of MDSCs on immune cells. Regulatory T cells (Tregs) also play a key role in maintaining autoimmune tolerance. Insufficient Treg populations cause autoimmune disease due to the decline of autoimmune tolerance. However, in tumors, immune tolerance is strong due to excessive Treg abundance, which further inhibits the activity of immune cells and causes resistance to immunotherapy [53]. Possible mechanisms for this include the production of immunosuppressive cytokines, direct cell contact inhibition, and cell lysis [54]. In recent years, Akt inhibitors have shown promise in inhibiting the immunosuppressive effects of Tregs. Inhibition of Akt signaling can inhibit Tregs, reduce the inhibitory effect of Tregs on endogenous antitumor immunity, increase infiltration of CD8+ T cells, and enhance antitumor immunity. Furthermore, Tregs secrete IL-10 and chelated IL-2, which inhibit antitumor immune responses; therefore, Treg inhibition can promote antitumor immune responses by decreasing IL-10 and chelated IL-2 secretion [53, 55]. In a clinical trial of an Akt inhibitor, Ipatasertib, plus paclitaxel, mutations in PI3K-Akt pathway genes, such as *PIK3CA* and *AKT1*, could enhance the therapeutic effect of the Akt inhibitor [56]. Mutations in PI3K reduce the expression of Cyclin D1 and cyclin-dependent kinase 4 (Cdk4), a multiprotein structure that is essential for the transition from G1 to S phase of the cell cycle.

This study was not without limitations. Data from the immunotherapy cohort was targeted sequencing data, which is less thorough than WES; therefore, some mutations in other genes of the PI3K/Akt pathway may have been missed. Furthermore, because the immunotherapy cohort for COAD was very limited, we only used publicly available data; in future studies, we will select additional immunotherapy cohorts for validation. Our study was also limited by the lack of corresponding animal and cell line experiments.

In the present study, we studied the effect of PI3K/Akt pathway mutations on the prognosis of COAD patients and speculated on the corresponding mechanisms. Future research should confirm our findings in additional immunotherapy cohorts as well as in cell line and animal experiments. It is also necessary to conduct experiments at the molecular level to determine the mechanism by which PI3K/Akt pathway mutations improve the prognosis of COAD patients.

## 5. Conclusions

The present study found that PI3K/Akt pathway mutations are an independent predictor of improved prognosis for COAD patients receiving ICI treatments. Patients in the PI3K/Akt-MT group had significantly prolonged OS compared to patients in the PI3K/Akt-WT group. In addition, the PI3K-Akt-MT group had significantly increased immunogenicity, significantly enriched immune cell infiltration, and changes in the expression of immune markers. Changes in the immune microenvironment were conducive to inhibiting the growth and migration of cancer cells. PI3K/Akt mutation status can therefore be used as a novel predictor of ICI response in COAD patients.

## Data Availability

The datasets generated during and/or analyzed during the current study are available from the corresponding authors on reasonable request.

## Abbreviations

ICIs: Immune checkpoint inhibitors
COAD: Colon adenocarcinoma
TCGA: The Cancer Genome Atlas
WES: Whole-exome sequencing
GSEA: Gene set enrichment analysis
KM: Kaplan-Meier
OS: Overall survival
PI3K/Akt-MT: PI3K/Akt-mutated type
PI3K/Akt-WT: PI3K/Akt-wild type
TMB: Tumor mutation burden
NAL: Neoantigen load
CTLA4: Cytotoxic T lymphocyte-associated protein-4
PD-L1: Programmed cell death-ligand 1
BER: Base excision repair
MMR: Mismatch repair
SSB: Single-stranded breaks
DSB: Double-stranded breaks
NER: Nucleotide excision repair
NHEJ: Nonhomologous end joining
FA: Fanconi anemia
KEGG: Kyoto Encyclopedia of Genes and Genomes
TCR: T cell receptor
DDR: DNA damage repair.

## Supplementary Materials

Supplementary Materials Supplementary Figure 1: mutual exclusion analysis of the top 20 mutant genes in the immunotherapy cohort (a) and TCGA cohort (b) (^∗^*P* < 0.05; ^∗∗^*P* < 0.01; ^∗∗∗^*P* < 0.001; ^∗∗∗∗^*P* < 0.0001; Fisher's exact test). Table S1: the immunogenic characteristics of patients in Local-COAD. Table S2: the list of detected genes in targeted sequencing.
