# Supplementary material for: Comprehensive Analysis Identifies PI3K/Akt Pathway Alternations as an Immune-Related Prognostic Biomarker in Colon Adenocarcinoma Patients Receiving Immune Checkpoint Inhibitor Treatment
Source: J Immunol Res. 2022 Jun 6;2022:8179799. doi: 10.1155/2022/8179799 (PMC9192307; doi:10.1155/2022/8179799)
Supplement: Supplementary Materials — Supplementary Figure 1: mutual exclusion analysis of the top 20 mutant genes in the immunotherapy cohort (a) and TCGA cohort (b) (∗P < 0.05; ∗∗P < 0.01; ∗∗∗P < 0.001; ∗∗∗∗P < 0.0001; Fisher's exact test). Table S1: the immunogenic characteristics of patients in Local-COAD. Table S2: the list of detected genes in targeted sequencing. [file 8179799.f1.zip › Supplementary Method.docx]

Supplementary Methods

Sample collection and targeted sequencing

This study has been approved by the Ethics Committee of Zhujiang Hospital of Southern Medical University and informed consent of all participating patients. We collected 50 COAD patients from Zhujiang Hospital of Southern Medical University. Of the 50 patients, 50 formalin-fixed paraffin-embedded (FFPE) tumor samples were also collected. We used HapOncoTM Solid Tumor Clinical 680 Gene Panel to conduct targeted sequencing of COAD patients. The genes involved in targeted sequencing were detailed in Supplementary Table 2. The generated library was sequenced on NovaSeq6000 (Illumina, San Diego, CA, USA), according to PE150 strategies.
