# Supplementary material for: Comprehensive Analysis Identifies PI3K/Akt Pathway Alternations as an Immune-Related Prognostic Biomarker in Colon Adenocarcinoma Patients Receiving Immune Checkpoint Inhibitor Treatment
Source: J Immunol Res. 2022 Jun 6;2022:8179799. doi: 10.1155/2022/8179799 (PMC9192307; doi:10.1155/2022/8179799)
Supplement: Supplementary Materials — Supplementary Figure 1: mutual exclusion analysis of the top 20 mutant genes in the immunotherapy cohort (a) and TCGA cohort (b) (∗P < 0.05; ∗∗P < 0.01; ∗∗∗P < 0.001; ∗∗∗∗P < 0.0001; Fisher's exact test). Table S1: the immunogenic characteristics of patients in Local-COAD. Table S2: the list of detected genes in targeted sequencing. [file 8179799.f1.zip › Table S1.pdf]

	PI3K/AKT signaling-MT (N=26)	PI3K/AKT signaling-WT (N=24)	Overall (N=50)
**MSI Status**			
MSI-H	5 (19.2%)	0 (0%)	5 (10.0%)
MSS/MSI-L	21 (80.8%)	24 (100%)	45 (90.0%)
**PDL1 TPS**			
TPS<1%	18 (69.2%)	18 (75.0%)	36 (72.0%)
TPS=1%	4 (15.4%)	2 (8.3%)	6 (12.0%)
TPS=2%	1 (3.8%)	0 (0%)	1 (2.0%)
TPS=5%	1 (3.8%)	0 (0%)	1 (2.0%)
Missing	2 (7.7%)	4 (16.7%)	6 (12.0%)
**PDL1 CPS**			
CPS<1%	13 (50.0%)	17 (70.8%)	30 (60.0%)
CPS=1%	7 (26.9%)	3 (12.5%)	10 (20.0%)
CPS=2%	2 (7.7%)	0 (0%)	2 (4.0%)
CPS=3%	1 (3.8%)	0 (0%)	1 (2.0%)
CPS=5%	1 (3.8%)	0 (0%)	1 (2.0%)
Missing	2 (7.7%)	4 (16.7%)	6 (12.0%)
**TMB**			
Mean (SD)	18.6 (28.0)	2.88 (1.73)	11.1 (21.6)
Median [Min, Max]	5.59 [0, 99.3]	3.29 [0, 5.92]	3.95 [0, 99.3]
