# Supplementary material for: Comprehensive Analysis Identifies PI3K/Akt Pathway Alternations as an Immune-Related Prognostic Biomarker in Colon Adenocarcinoma Patients Receiving Immune Checkpoint Inhibitor Treatment
Source: J Immunol Res. 2022 Jun 6;2022:8179799. doi: 10.1155/2022/8179799 (PMC9192307; doi:10.1155/2022/8179799)
Supplement: Supplementary Materials — Supplementary Figure 1: mutual exclusion analysis of the top 20 mutant genes in the immunotherapy cohort (a) and TCGA cohort (b) (∗P < 0.05; ∗∗P < 0.01; ∗∗∗P < 0.001; ∗∗∗∗P < 0.0001; Fisher's exact test). Table S1: the immunogenic characteristics of patients in Local-COAD. Table S2: the list of detected genes in targeted sequencing. [file 8179799.f1.zip › Table S2.pdf]

Schedule 1: Solid Tumor Clinical 680 Genes Full List

ABCB1 (NM_000927)
ACTL6A (NM_004301)
ALDH2 (NM_000690)
AREG (NM_001657)
ATR (NM_001184)
ABCC1 (NM_004996)
ACVR1 (NM_001105)
ALK (NM_004304)
ATRIP (NM_130384)
ARID1A (NM_006015)
ABCC11 (NM_032583)
ADCY2 (NM_020546)
ALOX12 (NM_000697)
ARID1B (NM_020732)
ATRX (NM_000489)
ABCC2 (NM_000392)
ADH1B (NM_000668)
AMER1 (NM_152424)
ARID2 (NM_152641)
AURKA (NM_003600)
ABCC4 (NM_005845)
ADH1C (NM_000669)
ANXA5 (NM_001154)
AURKB (NM_004217)
ARMS2 (NM_001099667)
ABCC5 (NM_005688)
AIP (NM_003977)
APC (NM_000038)
ASNS (NM_001673)
AXIN1 (NM_003502)
ABCG1 (NM_016818)
AXIN2 (NM_004655)
APLF (NM_173545)
ASPH (NM_004318)
AKR1C3 (NM_001253908)
ABCG2 (NM_004827)
AKT1 (NM_005163)
APOB (NM_000384)
ASXL1 (NM_015338)
AXL (NM_001699)
ABL1 (NM_005157)
AKT2 (NM_001626)
AR (NM_000044)
ATM (NM_000051)
ACSS2 (NM_018677)
AKT3 (NM_181690)
ARAF (NM_001654)
ATP7B (NM_000053)
B2M (NM_004048)
BCL2L1 (NM_138578)

BCORL1 (NM_021946)
BRCA1 (NM_007294)
BRE (NM_004899)
BAP1 (NM_004656)
BCL2L11 (NM_138625)
BLM (NM_000057)
BRCA2 (NM_000059)
BRIP1 (NM_032043)
BARD1 (NM_000465)
BCL6 (NM_001706)
BMPR1A (NM_004329)
BRD2 (NM_001113182)
BTK (NM_000061)
BCL2 (NM_000633)
BCOR (NM_001123383)
BRAF (NM_004333)
BRD4 (NM_058243)
BUB1 (NM_004336)
C8orf34 (NM_052958)
CCL18 (NM_002988)
CDK8 (NM_001260)
CLK2 (NM_003993)
CXXC4 (NM_025212)
CACNA1C (NM_000719)
CCND1 (NM_053056)
CDKN1A (NM_078467)
CMPK1 (NM_016308)
CYLD (NM_015247)
CADM2 (NM_153184)
CCND2 (NM_001759)
CDKN1B (NM_004064)
CYP19A1 (NM_000103)
CNTNAP5 (NM_130773)
CALR (NM_004343)
CCND3 (NM_001760)
CDKN1C (NM_000076)
CREBBP (NM_004380)
CYP1A1 (NM_000499)
CAMTA1 (NM_015215)
CCNE1 (NM_001238)
CDKN2A (NM_000077)
CRKL (NM_005207)
CYP1A2 (NM_000761)
CAPN2 (NM_001748)
CD274 (NM_014143)
CDKN2B (NM_004936)
CRLF2 (NM_022148)
CYP1B1 (NM_000104)
CARD11 (NM_032415)
CD74 (NM_001025159)
CDKN2C (NM_001262)

CSDE1 (NM_001007553)
CYP2B6 (NM_000767)
CASC8 (NR_024393)
CD79A (NM_001783)
CEBPA (NM_004364)
CSF1R (NM_005211)
CYP2C19 (NM_000769)
CASP7 (NM_001227)
CD79B (NM_001039933)
CES1 (NM_001025195)
CSF3R (NM_156039)
CYP2C8 (NM_000770)
CASP8 (NM_001228)
CDA (NM_001785)
CFD (NM_001928)
CSMD3 (NM_052900)
CYP2C9 (NM_000771)
CBFB (NM_001755)
CDC25C (NM_001790)
CFH (NM_000186)
CSNK1A1 (NM_001892)
CYP2D6 (NM_000106)
CBL (NM_005188)
CDC73 (NM_024529)
CFTR (NM_000492)
CSNK2A1 (NM_001895)
CYP2E1 (NM_000773)
CBLB (NM_170662)
CDH1 (NM_004360)
CHD4 (NM_001273)
CTCF (NM_006565)
CYP3A4 (NM_017460)
CBR1 (NM_001757)
CDK12 (NM_016507)
CHEK1 (NM_001274)
CTNNA1 (NM_001903)
CYP3A5 (NM_000777)
CBR3 (NM_001236)
CDK2 (NM_001798)
CHEK2 (NM_007194)
CTNNB1 (NM_001904)
CCAT2 (NR_109834)
CDK4 (NM_000075)
FNTB (NM_002028.3)
CUL3 (NM_003590)
CCDC6 (NM_005436)
CDK6 (NM_001259)
CIC (NM_015125)
CXCR4 (NM_003467)
DAXX (NM_001141969)
DDR2 (NM_006182)

DHFR (NM_000791)
DNMT3A (NM_022552)
DSCAM (NM_001389)
DDB2 (NM_000107)
DDX3X (NM_001356)
DICER1 (NM_177438)
DOT1L (NM_032482)
DYNC2H1 (NM_001080463)
DDIT3 (NM_004083)
DDX51 (NM_175066)
DNMT1 (NM_001130823)
DPYD (NM_000110)
E2F7 (NM_203394)
EML4 (NM_019063)
EPHB1 (NM_004441)
ERCC5 (NM_000123)
ETV6 (NM_001987)
ECT2L (NM_001077706)
ENOSF1 (NM_017512)
EPHX1 (NM_001136018)
ERCC6 (NM_000124)
EWSR1 (NM_005243)
EDN1 (NM_001955)
EP300 (NM_001429)
ERBB2 (NM_004448)
EREG (NM_001432)
EXT1 (NM_000127)
EED (NM_003797)
EPAS1 (NM_001430)
ERBB3 (NM_001982)
ERG (NM_182918)
EXT2 (NM_000401)
EEF1A1 (NM_001402)
EPCAM (NM_002354)
ERBB4 (NM_001042599)
ERRFI1 (NM_018948)
EZH2 (NM_004456)
EGF (NM_001963)
EPHA2 (NM_004431)
ERCC1 (NM_001983)
ESR1 (NM_000125)
EGFR (NM_005228)
EPHA3 (NM_005233)
ERCC2 (NM_000400)
ESR2 (NM_001040275)
EIF3A (NM_003750)
EPHA5 (NM_004439)
ERCC3 (NM_000122)
ETV1 (NM_004956)
ELAC2 (NM_001165962)
EPHA7 (NM_004440)

ERCC4 *(NM_005236)*
ETV4 *(NM_001986)*
FAM175A (NM_139076)
FAT1 (NM_005245)
FGF23 (NM_020638)
FGFR2 (NM_000141)
FOXA1 (NM_004496)
FAM46C (NM_017709)
FBN3 (NM_032447)
FGF3 (NM_005247)
FGFR3 (NM_000142)
FOXK2 (NM_004514)
FANCA (NM_000135)
FBXW7 (NM_018315)
FGF4 (NM_002007)
FGFR4 (NM_022963)
FOXL2 (NM_023067)
FANCB (NM_001018113)
FCGR2A (NM_021642)
FGF5 (NM_004464)
FH (NM_000143)
FOXM1 (NM_021953)
FANCC (NM_000136)
FCGR3A (NM_001127593)
FGF6 (NM_020996)
FLCN (NM_144997)
FOXP1 (NM_032682)
FANCG (NM_004629)
FGF1 (NM_000800)
FGF7 (NM_002009)
FLT1 (NM_002019)
FOXP2 (NM_014491)
FANCI (NM_001113378)
FGF10 (NM_004465)
FGF8 (NM_006119)
FLT3 (NM_004119)
FRS2 (NM_006654)
FANCL (NM_018062)
FGF19 (NM_005117)
FGF9 (NM_002010)
FLT4 (NM_182925)
FUBP1 (NM_003902)
FANCM (NM_020937)
FGF2 (NM_002006)
FGFR1 (NM_023110)
FOLR3 (NM_000804)
FUS (NM_004960)
GAB2 (NM_080491)
GATA6 (NM_005257)
GLIPR1 (NM_006851)
GPER1 (NM_001039966)

GSK3B (NM_001146156)
GALNT12 (NM_024642)
GEMIN6 (NM_024775)
GLRX (NM_002064)
GPRIN2 (NM_014696)
GSR (NM_000637)
GALNT14 (NM_001253826) *GEN1* (NM_182625)
GMEB1 (NM_006582)
GPX5 (NM_001509)
GSTA1 (NM_145740)
GATA1 (NM_002049)
GGH (NM_003878)
GNA11 (NM_002067)
GREM1 (NM_013372)
GSTM3 (NM_000849)
GATA2 (NM_032638)
GK5 (NM_001039547)
GNAQ (NM_002072)
GRIN2A (NM_000833)
GSTP1 (NM_000852)
GATA3(NM_001002295)
GLI1(NM_005269)
GNAS(NM_000516)
GRM1(NM_001278064)
H19 (NR_002196)
HGF (NM_000601)
HLA-DPB1 (NM_002121)
HNF1A (NM_000545)
HSD17B3 (NM_000197)
H3F3A (NM_002107)
HIF1A (NM_001530)
HLA-DQB1 (NM_002123)
HNF1B (NM_000458)
HSD3B2 (NM_000198)
HDAC2 (NM_001527)
HKDC1 (NM_025130)
HLA-DRB1 (NM_002124)
HOTAIR (NR_047517)
HSP90AA1 (NM_005348)
HERC2 (NM_004667)
HLA-A (NM_002116)
HLA-G (NM_002127)
HOXB13 (NM_006361)
HSPA5 (NM_005347)
HFE2 (NM_145277)
HLA-B (NM_005514)
HMGA2 (NM_003483)
HPSE2 (NM_021828)
HTRA1 (NM_002775)
HFM1 (NM_001017975)
HLA-C (NM_002117)

HMGCR (NM_000859)
HRAS (NM_005343)
IDH1 (NM_005896)
IFNLR1 (NM_170743)
IKZF1 (NM_006060)
IL7R (NM_002185)
IRS2 (NM_003749)
IDH2 (NM_002168)
IGF1R (NM_000875)
IL13 (NM_002188)
INHBA (NM_002192)
JAK1 (NM_002227)
IFNGR1 (NM_000416)
IGF2 (NM_000612)
IL16 (NM_001172128)
INPP4B (NM_003866)
JAK2 (NM_004972)
IFNGR2 (NM_005534)
IGFBP3 (NM_001013398)
IL1B (NM_000576)
*IRF*1 (NM_002198)
JAK3 (NM_000215)
IFNL2 (NM_172138)
IKBKE (NM_001193321)
IL23R (NM_144701)
IRF4 (NM_001195286)
JUN (NM_002228)
KCNJ5 (NM_000890)
KDR (NM_002253)
KIT (NM_000222)
KMT2A (NM_001197104)
KRAS (NM_004985)
KDM5A (NM_001042603) *KEAP1* (NM_012289)
KLC1 (NM_001130107)
KMT2B (NM_014727)
KRT14 (NM_000526)
KDM5C (NM_004187)
KIF1B (NM_015074)
KLF4 (NM_004235)
KMT2C (NM_170606)
KRT15 (NM_002275)
KDM6A (NM_021140)
KIF5B (NM_004521)
KLLN (NM_001126049)
KMT2D (NM_003482)
KRT5 (NM_000424)
LARP4 (NM_052879)
LBR (NM_002296)
LIG3 (NM_013975)
LRIG3 (NM_153377)
LRP2 (NM_004525)

LATS1 (NM_004690)
LGALS8 *(NM_006499)*
LIG4 *(NM_002312)*
LRMDA *(NM_032024.3)*
LYN *(NM_002350)*
LATS2 (NM_014572)
LGR5 *(NM_003667)*
LMO1 *(NM_002315)*
LRP1B *(NM_018557)*
LZTR1 *(NM_006767)*
MAD1L1 (NM_003550)
MAPK3 *(NM_001040056)*
MEN1 *(NM_000244)*
MPL *(NM_005373)*
MUTYH *(NM_001128425)*
MALAT1 (NR_002819)
MAPKBP1 *(NM_014994)*
MET *(NM_001127500)*
MRE11A *(NM_005590)*
MYC *(NM_002467)*
MAP2K1 (NM_002755)
MAX *(NM_002382)*
MGAT4A *(NM_012214)*
MSH2 *(NM_000251)*
MYCL *(NM_001033082)*
MAP2K2 (NM_030662)
MCL1 *(NM_021960)*
MIR3936HG *(NR_110997)*
MSH3 *(NM_002439)*
MYCN *(NM_005378)*
MAP2K4 (NM_003010)
MDH2 *(NM_005918)*
MIR4713HG *(NR_146310)*
MSH6 *(NM_000179)*
MYD88 *(NM_001172567)*
MAP3K1 (NM_005921)
MDM2 *(NM_002392)*
MITF *(NM_000248)*
MST1R *(NM_001244937)*
MYOD1 *(NM_002478)*
MAP3K6 (NM_004672)
MDM4 *(NM_002393)*
MKI67 *(NM_002417)*
MTHFR *(NM_005957)*
MAP4K4 (NM_004834)
MED12 *(NM_005120)*
MLH1 *(NM_000249)*
MTOR *(NM_004958)*
MAPK1 (NM_002745)
MEF2B *(NM_005919)*
MLH3 *(NM_001040108)*

MTUS1 *(NM_020749)*
NAB2 (NM_005967)
NF2 *(NM_000268)*
NOTCH3 *(NM_000435)*
NRAS *(NM_002524)*
NTRK3 *(NM_001007156)*
NAT2 (NM_000015)
NFE2L2 *(NM_006164)*
NOVA1 *(NM_006491)*
NRG1 *(NM_001159995)*
NUP93 *(NM_014669)*
NBN (NM_002485)
NFKBIA *(NM_020529)*
NPM1 *(NM_002520)*
NSD1 *(NM_022455)*
NUTM1 *(NM_175741)*
NCOA1 (NM_003743)
NKX2-1 *(NM_003317)*
NQO1 *(NM_000903)*
NT5C2 *(NM_012229)*
OPRM1 (NM_001008503)
NCOA3 (NM_006534)
NOS3 *(NM_000603)*
NQO2 *(NM_000904)*
NTHL1 *(NM_002528)*
OTOS *(NM_148961)*
NCOA4 (NM_001145260) *NOTCH1* *(NM_017617)*
NR1I2 *(NM_003889)*
NTRK1 *(NM_002529)*
NF1(NM_000267)
NOTCH2(NM_024408)
NR4A3(NM_006981)
NTRK2(NM_006180)
PAK1 (NM_002576)
PCLO *(NM_014510)*
PIK3CG *(NM_002649)*
POR *(NM_000941)*
PRSS1 *(NM_002769)*
PALB2 (NM_024675)
PDCD1 *(NM_005018)*
PIK3R1 *(NM_181523)*
PPIB *(NM_000942)*
PSME2 *(NM_002818)*
PALLD (NM_016081)
PDCD1LG2 *(NM_025239)*
PIK3R2 *(NM_005027)*
PPP2R1A *(NM_014225)*
PTCH1 *(NM_000264)*
PARD3B *(NM_057177)*
PDGFB *(NM_002608)*
PIM1 *(NM_001243186)*

PPP2R2A *(NM_002717)*
PTCH2 *(NM_003738)*
PARK2 (NM_004562)
PDGFRA (NM_006206)
PLAUR (NM_002659)
PPP4R4 (NM_058237)
PTEN (NM_000314)
PARK7 (NM_007262)
PDGFRB (NM_002609)
PLCG2 (NM_002661)
PRDM1 (NM_001198)
PTGER4 (NM_000958)
PARP1 (NM_001618)
PDPK1 (NM_002613)
PLIN2 (NM_001122)
PRDX4 (NM_006406)
PTGES (NM_004878)
PAX3 (NM_000438)
PGR (NM_000926)
PMS1 (NM_000534)
PREX2 (NM_025170)
PTGS2 (NM_000963)
PAX5 (NM_016734)
PIAS4 (NM_015897)
PMS2 (NM_000535)
PRKACA (NM_002730)
PTN (NM_002825)
PAX7 (NM_002584)
PIGB (NM_004855)
POLD1 (NM_002691)
PRKACB (NM_182948)
PTPN11 (NM_002834)
PAX8 (NM_003466)
PIK3CA (NM_006218)
POLE (NM_006231)
PRKAR1A (NM_002734)
PTPRD (NM_001040712)
PBRM1 (NM_018165)
PIK3CB (NM_006219)
POLH (NM_006502)
PRKCI (NM_002740)
PTPRT (NM_007050)
PCBP1 (NM_006196)
PIK3CD (NM_005026)
POLQ (NM_199420)
PRKDC (NM_006904)
PZP (NM_002864)
RAC1 (NM_018890)
RAF1 (NM_002880)
RET (NM_020630)
RIF1 (NM_001177663)

RPS20 (NM_001023)
RAD21 (NM_006265)
RARA (NM_000964)
REV3L (NM_002912)
RILP (NM_031430)
RPS6KB1 (NM_003161)
RAD50 (NM_005732)
RB1 (NM_000321)
RFC1 (NM_002913)
RINT1 (NM_021930)
RPTOR (NM_020761)
RAD51 (NM_002875)
RBFOX1 (NM_145891)
RFC4 (NM_002916)
RIT1 (NM_006912)
RRAS2 (NM_001177314)
RAD51B (NM_002877)
RBM10 (NM_005676)
RGS5 (NM_003617)
RNASEL (NM_021133)
RRM1 (NM_001033)
RAD51C (NM_058216)
RECK (NM_021111)
RHBDF2 (NM_024599)
RNF43 (NM_017763)
RSF1 (NM_016578)
RAD51D (NM_002878)
RECQL (NM_002907)
RHEB (NM_005614)
ROBO2 (NM_002942)
RUNX1 (NM_001754)
RAD52 (NM_134424)
RECQL4 (NM_004260)
RHOA (NM_001664)
ROS1 (NM_002944)
RAD54L (NM_003579)
REL (NM_002908)
RICTOR (NM_152756)
RPA4 (NM_013347)
SBDS (NM_016038)
SETD2 (NM_014159)
SLC22A5 (NM_003060)
SMARCB1 (NM_003073)
SRD5A2 (NM_000348)
SCN10A (NM_006514)
SETD7 (NM_030648)
SLC28A1 (NM_004213)
SMO (NM_005631)
SRSF2 (NM_003016)
SDHA (NM_004168)
SF3B1 (NM_012433)

SLC28A2 (NM_004212)
SOCS1 (NM_003745)
SS18 (NM_001007559)
SDHAF2 (NM_017841)
SH2B3 (NM_005475)
SLC29A1 (NM_004955.2)
SOCS6 (NM_004232)
STAG2 (NM_001042749)
SDHB (NM_003000)
SHMT1 (NM_004169)
SLC31A1 (NM_001859)
SOD2 (NM_001024465)
STAT3 (NM_139276)
SDHC (NM_003001)
SHOX (NM_000451)
SLC34A2 (NM_006424)
SOX10 (NM_006941)
STK11 (NM_000455)
SDHD (NM_003002)
SHPRH (NM_173082)
SLCO1B1 (NM_006446)
SOX2 (NM_003106)
SUFU (NM_016169)
SELE (NM_000450)
SLC15A2 (NM_021082)
SLCO1B3 (NM_019844)
SOX4 (NM_003107)
SULT1A1 (NM_001055)
SELL (NM_000655)
SLC19A1 (NM_030582)
SLX4 (NM_032444)
SOX9 (NM_000346)
SUZ12 (NM_015355)
SEMA3C (NM_006379)
SLC22A1 (NM_003057)
SMAD2 (NM_005901)
SPEN (NM_015001)
SWI5 (NM_001040011)
SERPINB3 (NM_006919)
SLC22A16 (NM_033125)
SMAD3 (NM_005902)
SPINK1 (NM_003122)
SYK (NM_001135052)
S ERPINB4 (NM_002974)
SLC22A2 (NM_003058)
SMAD4 (NM_005359)
SPOP (NM_003563)
SYNE1 (NM_033071)
SETBP1 (NM_015559)
SLC22A4 (NM_003059)
SMARCA4 (NM_001128849) *SRC* (NM_198291)

TACSTD2 (NM_002353)
TFE3 (NM_006521)
TNFRSF11B (NM_002546) *TP53* (NM_000546)
TSC2 (NM_000548)
TBX3 (NM_005996)
TGFB1 (NM_000660)
TNFRSF14 (NM_003820)
TP53BP1 (NM_001141979) *TSHR* (NM_000369)
TCEB1 (NM_005648)
TGFBR2 (NM_001024847)
TNFRSF19 (NM_018647)
TPMT (NM_000367)
TSPAN31 (NM_005981)
TERC (NR_001566)
TMEM127 (NM_017849)
TNFSF11 (NM_033012)
TRAF1 (NM_005658)
TTK (NM_001166691)
TERT (NM_198253)
TMPRSS2 (NM_001135099) *TNFSF8* (NM_001244)
TRAM2-AS1 (NR_103446) *TUBB1* (NM_030773)
TET1 (NM_030625)
TNF (NM_000594)
TOP1 (NM_003286)
TREX2 (NM_080701)
TYMS (NM_001071)
TET2 (NM_017628)
TNFAIP3 (NM_006290)
TOP2A (NM_001067)
TSC1 (NM_000368)
U2AF1 (NM_006758)
UBE2A (NM_003336)
UBE2I (NM_003345)
UBE2V2 (NM_003350)
UGT1A1 (NM_000463)
UGT1A4 (NM_007120)
UGT1A6 (NM_001072)
UGT1A9 (NM_021027)
VEGFA (NM_001025366)
VEGFC (NM_005429)
VHL (NM_000551)
WAS (NM_000377)
WIF1 (NM_007191)
WNK2 (NM_006648)
WNT5B (NM_032642)
WRN (NM_000553)
WT1 (NM_024426)
XBP1 (NM_005080)
XPA (NM_000380)
XPC (NM_004628)
XPO1 (NM_003400)

XRCC1 (NM_006297)
XRCC2 (NM_005431)
XRCC4 (NM_022406)
YAP1 *(NM_006106)*
YES1 (NM_005433)
ZBTB16 (NM_006006)
ZNF367 (NM_153695)
ZNF423 (NM_001271620)
ZNF750 (NM_024702)
ZRSR2 (NM_005089)

Note: The detection range covers the entire coding region of 528 genes and the adjacent introns ± 20bp, and part of the region of 152 genes. Gene names followed the rules of the HUGO Gene Nomenclature Committee (HGNC); gene transcripts were selected using the Locus Reference Genomic.

Schedule 2: List of fusion genes
ALK (NM_004304)
FGFR1 (NM_023110)
NAB2 (NM_005967)
NTRK3 (NM_001007156)
RARA (NM_000964)
BRAF (NM_004333)
FGFR2 (NM_000141)
NCOA4 (NM_001145260)
NUTM1 (NM_175741)
RET (NM_020630)
CCDC6 (NM_005436)
FGFR3 (NM_000142)
NPM1 (NM_002520)
PAX3 (NM_000438)
ROS1 (NM_002944)
CD74 (NM_001025159)
FUS (NM_004960)
NR4A3 (NM_006981)
PAX7 (NM_002584)
SLC34A2 (NM_006424)
DDIT3 (NM_004083)
KIF5B (NM_004521)
NRG1 (NM_001159995)
PAX8 (NM_003466)
SS18 (NM_001007559)
EML4 (NM_019063)
KMT2A (NM_001197104)
NTRK1 (NM_002529)
PDGFB (NM_002608)
TFE3 (NM_006521)
EWSR1 (NM_005243)
MYC (NM_002467)

NTRK2 (NM_006180)
PRKACA (NM_002730)
TMPRSS2 (NM_001135099)

Note: The fusion gene covers the entire coding region and the fusion intron region, and some genes cover the fusion partner fusion intron region.

Schedule 3 List of hereditary tumor-related genes
AIP (NM_003977)
CDKN2A (NM_000077)
HOXB13 (NM_006361)
POLD1 (NM_002691)
SDHB (NM_003000)
ALK (NM_004304)
CFTR (NM_000492)
MAX (NM_002382)
POLE (NM_006231)
SDHC (NM_003001)
APC (NM_000038)
CHEK2 (NM_007194)
MDH2 (NM_005918)
PRKAR1A (NM_002734)
SDHD (NM_003002)
ATM (NM_000051)
CTNNA1 (NM_001903)
MEN1 (NM_000244)
PRSS1 (NM_002769)
SLX4 (NM_032444)
ATR (NM_001184)
DICER1 (NM_177438)
MET (NM_001127500)
PTCH1 (NM_000264)
SMAD4 (NM_005359)
AXIN2 (NM_004655)
EPAS1 (NM_001430)
MLH1 (NM_000249)
PTEN (NM_000314)
SMARCA4 (NM_001128849)
BAP1 (NM_004656)
EPCAM (NM_002354)
MLH3 (NM_001040108)
RAD50 (NM_005732)
SMARCB1 (NM_003073)
BARD1 (NM_000465)
EXT1 (NM_000127)
MRE11A (NM_005590)
RAD51C (NM_058216)
SPINK1 (NM_003122)
BLM (NM_000057)
EXT2 (NM_000401)

MSH2 (NM_000251)
RAD51D (NM_002878)
STK11 (NM_000455)
BMPR1A (NM_004329)
FAM175A (NM_139076)
MSH3 (NM_002439)
RB1 (NM_000321)
SUFU (NM_016169)
BRCA1 (NM_007294)
FANCA (NM_000135)
MSH6 (NM_000179)
RECQL4 (NM_004260)
TERT (NM_198253)
BRCA2 (NM_000059)
FANCC (NM_000136)
MUTYH (NM_001128425)
RET (NM_020630)
TMEM127 (NM_017849)
BRIP1 (NM_032043)
FANCG (NM_004629)
NBN (NM_002485)
RHBDF2 (NM_024599)
TP53 (NM_000546)
CDC73 (NM_024529)
FH (NM_000143)
NF1 (NM_000267)
RINT1 (NM_021930)
TSC1 (NM_000368)
CDH1 (NM_004360)
FLCN (NM_144997)
NF2 (NM_000268)
RNF43 (NM_017763)
TSC2 (NM_000548)
CDK12 (NM_016507)
GALNT12 (NM_024642)
NTHL1 (NM_002528)
RPS20 (NM_001023)
VHL (NM_000551)
CDK4 (NM_000075)
GEN1 (NM_182625)
PALB2 (NM_024675)
SDHA (NM_004168)
XRCC2 (NM_005431)
CDKN1B (NM_004064)
GREM1 (NM_013372)
PMS2 (NM_000535)
SDHAF2 (NM_017841)

Note: The detection of hereditary tumor-related genes covers the entire coding region and adjacent introns ± 20bp, and also covers 387 related databases that have reported possible pathogenic intron and regulatory region mutations.

Schedule 4 List of DNA Damage Repair (DDR) Related Genes

Homologous recombination repair (HRR) pathway related genes

ATM (NM_000051)
CHEK1 (NM_001274)
FANCI (NM_001113378)
PALB2 (NM_024675)
RAD52 (NM_134424)
ATR (NM_001184)
CHEK2 (NM_007194)
FANCL (NM_018062)
RAD50 (NM_005732)
RAD54L (NM_003579)
BRCA1 (NM_007294)
FANCA (NM_000135)
FANCM (NM_020937)
RAD51 (NM_002875)
BARD1 (NM_000465)
BRCA2 (NM_000059)
FANCB (NM_001018113)
GEN1 (NM_182625)
RAD51B (NM_002877)
BRIP1 (NM_032043)
FANCC (NM_000136)
MRE11A (NM_005590)
RAD51C (NM_058216)
CDK12 (NM_016507)
FANCG (NM_004629)
NBN (NM_002485)
RAD51D (NM_002878)

DNA mismatch repair (MMR) pathway related genes
MLH1 (NM_000249)
MLH3 (NM_001040108)
MSH2 (NM_000251)
MSH3 (NM_002439)
MSH6 (NM_000179)
PMS1 (NM_000534)
PMS2 (NM_000535)

Nucleotide excision repair (NER) pathway related genes

ERCC1 (NM_001983)
ERCC2 (NM_000400)
ERCC3 (NM_000122)
ERCC4 (NM_005236)
ERCC5 (NM_000123)
ERCC6 (NM_000124)

Other DNA damage repair (DDR) related genes
ATRIP(NM_130384)
MUTYH (NM_001128425)
PRKDC (NM_006904)
RPA4 (NM_013347)
UBE2A (NM_003336)
BLM (NM_000057)
PARP1 (NM_001618)
RECQL (NM_002907)
SHPRH (NM_173082)
UBE2V2 (NM_003350)
CLK2 (NM_003993)
POLD1 (NM_002691)
RECQL4 (NM_004260)
TP53 (NM_000546)
WRN (NM_000553)
IDH1 (NM_005896)
POLE (NM_006231)
REV3L (NM_002912)
TP53BP1 (NM_001141979)
PTEN (NM_000314)
LIG4 (NM_002312)
POLQ (NM_199420)
RIF1 (NM_001177663)
TREX2 (NM_080701)
STAG2 (NM_001042749)

Note: DDR: DNA damage response; HRR: Homologous Recombination Repair ;MMR: DNA mismatch repair; NER: Nucleotide excision repair.

Schedule 2: List of fusion genes

ALK (NM_004304)
FGFR1 (NM_023110)
NAB2 (NM_005967)
NTRK3 (NM_001007156)
RARA (NM_000964)
BRAF (NM_004333)
FGFR2 (NM_000141)
NCOA4 (NM_001145260)
NUTM1 (NM_175741)
RET (NM_020630)
CCDC6 (NM_005436)
FGFR3 (NM_000142)
NPM1 (NM_002520)
PAX3 (NM_000438)
ROS1 (NM_002944)
CD74 (NM_001025159)
FUS (NM_004960)
NR4A3 (NM_006981)
PAX7 (NM_002584)
SLC34A2 (NM_006424)
DDIT3 (NM_004083)
KIF5B (NM_004521)
NRG1 (NM_001159995)
PAX8 (NM_003466)
SS18 (NM_001007559)
EML4 (NM_019063)
KMT2A (NM_001197104)
NTRK1 (NM_002529)
PDGFB (NM_002608)
TFE3 (NM_006521)
EWSR1 (NM_005243)
MYC (NM_002467)
NTRK2 (NM_006180)
PRKACA (NM_002730)
TMPRSS2 (NM_001135099)

Note: The fusion gene covers the entire coding region and the fusion intron region, and some genes c

over the fusion partner fusion intron region.

Schedule 3 List of hereditary tumor-related genes

AIP(NM_003977)
CDKN2A(NM_000077)
HOXB13(NM_006361)
POLD1(NM_002691)
SDHB(NM_003000)
ALK(NM_004304)
CFTR(NM_000492)
MAX(NM_002382)
POLE(NM_006231)
SDHC(NM_003001)
APC(NM_000038)
CHEK2(NM_007194)
MDH2(NM_005918)
PRKAR1A(NM_002734)
SDHD(NM_003002)
ATM(NM_000051)
CTNNA1(NM_001903)
MEN1(NM_000244)
PRSS1(NM_002769)
SLX4(NM_032444)
ATR(NM_001184)
DICER1(NM_177438)
MET(NM_001127500)
PTCH1(NM_000264)
SMAD4(NM_005359)
AXIN2(NM_004655)
EPAS1(NM_001430)
MLH1(NM_000249)
PTEN(NM_000314)
SMARCA4(NM_001128849)
BAP1(NM_004656)
EPCAM(NM_002354)
MLH3(NM_001040108)
RAD50(NM_005732)
SMARCB1(NM_003073)
BARD1(NM_000465)
EXT1(NM_000127)
MRE11A(NM_005590)
RAD51C(NM_058216)
SPINK1(NM_003122)
BLM(NM_000057)
EXT2(NM_000401)
MSH2(NM_000251)
RAD51D(NM_002878)
STK11(NM_000455)
BMPR1A(NM_004329)
FAM175A(NM_139076)
MSH3(NM_002439)
RB1(NM_000321)
SUFU(NM_016169)

BRCA1 (NM_007294)
FANCA (NM_000135)
MSH6 (NM_000179)
RECQL4 (NM_004260)
TERT (NM_198253)
BRCA2 (NM_000059)
FANCC (NM_000136)
MUTYH (NM_001128425)
RET (NM_020630)
TMEM127 (NM_017849)
BRIP1 (NM_032043)
FANCG (NM_004629)
NBN (NM_002485)
RHBDF2 (NM_024599)
TP53 (NM_000546)
CDC73 (NM_024529)
FH (NM_000143)
NF1 (NM_000267)
RINT1 (NM_021930)
TSC1 (NM_000368)
CDH1 (NM_004360)
FLCN (NM_144997)
NF2 (NM_000268)
RNF43 (NM_017763)
TSC2 (NM_000548)
CDK12 (NM_016507)
GALNT12 (NM_024642)
NTHL1 (NM_002528)
RPS20 (NM_001023)
VHL (NM_000551)
CDK4 (NM_000075)
GEN1 (NM_182625)
PALB2 (NM_024675)
SDHA (NM_004168)
XRCC2 (NM_005431)
CDKN1B (NM_004064)
GREM1 (NM_013372)
PMS2 (NM_000535)
SDHAF2 (NM_017841)
Note: The detection of hereditary tumor-related genes covers the entire coding region and adjacent in

trons ± 20bp, and also covers 387 related databases that have reported possible pathogenic intron and

regulatory region mutations.

Schedule 4 List of DNA Damage Repair (DDR) Related Genes

Homologous recombination repair (HRR) pathway related genes

ATM (NM_000051)
CHEK1 (NM_001274)
FANCI (NM_001113378)
PALB2 (NM_024675)
RAD52 (NM_134424)
ATR (NM_001184)
CHEK2 (NM_007194)
FANCL (NM_018062)
RAD50 (NM_005732)
RAD54L (NM_003579)
BRCA1 (NM_007294)
FANCA (NM_000135)
FANCM (NM_020937)
RAD51 (NM_002875)
BARD1 (NM_000465)
BRCA2 (NM_000059)
FANCB (NM_001018113)
GEN1 (NM_182625)
RAD51B (NM_002877)
BRIP1 (NM_032043)
FANCC (NM_000136)
MRE11A (NM_005590)
RAD51C (NM_058216)
CDK12 (NM_016507)
FANCG (NM_004629)
NBN (NM_002485)
RAD51D (NM_002878)

DNA mismatch repair (MMR) pathway related genes

MLH1 (NM_000249)
MLH3 (NM_001040108)
MSH2 (NM_000251)
MSH3 (NM_002439)
MSH6 (NM_000179)
PMS1 (NM_000534)
PMS2 (NM_000535)

Nucleotide excision repair (NER) pathway related genes

ERCC1 (NM_001983)
ERCC2 (NM_000400)
ERCC3 (NM_000122)
ERCC4 (NM_005236)
ERCC5 (NM_000123)
ERCC6 (NM_000124)

Other DNA damage repair (DDR) related genes

ATRIP (NM_130384)
MUTYH (NM_001128425)
PRKDC (NM_006904)
RPA4 (NM_013347)
UBE2A (NM_003336)
BLM (NM_000057)
PARP1 (NM_001618)
RECQL (NM_002907)
SHPRH (NM_173082)
UBE2V2 (NM_003350)
CLK2 (NM_003993)
POLD1 (NM_002691)
RECQL4 (NM_004260)
TP53 (NM_000546)
WRN (NM_000553)
IDH1 (NM_005896)
POLE (NM_006231)
REV3L (NM_002912)
TP53BP1 (NM_001141979)
PTEN (NM_000314)
LIG4 (NM_002312)
POLQ (NM_199420)
RIF1 (NM_001177663)
TREX2 (NM_080701)
STAG2 (NM_001042749)

Note: DDR: DNA damage response; HRR: Homologous Recombination Repair ;MMR: DNA misma

tch repair; NER:Nucleotide excision repair.
