# Supplementary figures and images for: Comprehensive Analysis Identifies PI3K/Akt Pathway Alternations as an Immune-Related Prognostic Biomarker in Colon Adenocarcinoma Patients Receiving Immune Checkpoint Inhibitor Treatment
Source: J Immunol Res. 2022 Jun 6;2022:8179799. doi: 10.1155/2022/8179799 (PMC9192307; doi:10.1155/2022/8179799)

**A**

ICI-treated

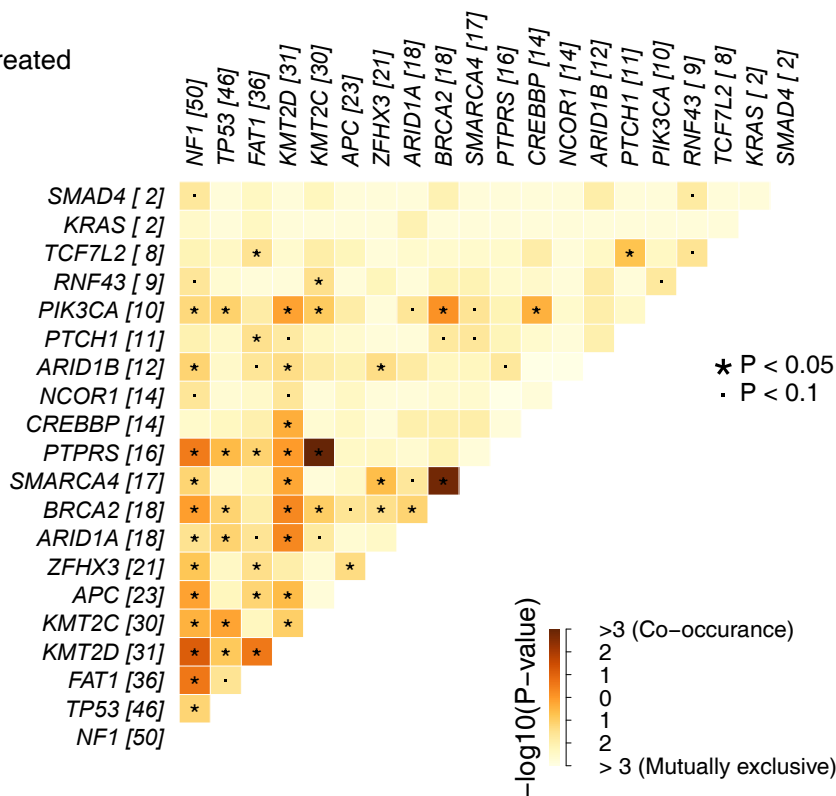**B**

TCGA-COAD

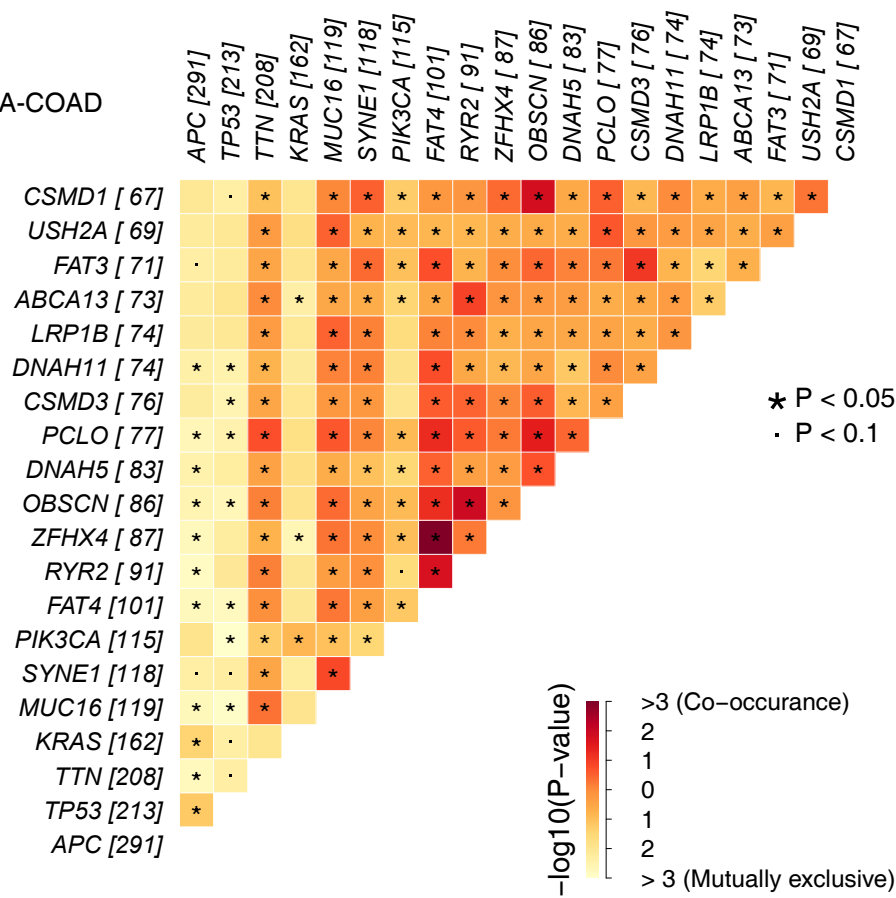

Supplement: Supplementary Materials — Supplementary Figure 1: mutual exclusion analysis of the top 20 mutant genes in the immunotherapy cohort (a) and TCGA cohort (b) (∗P < 0.05; ∗∗P < 0.01; ∗∗∗P < 0.001; ∗∗∗∗P < 0.0001; Fisher's exact test). Table S1: the immunogenic characteristics of patients in Local-COAD. Table S2: the list of detected genes in targeted sequencing. [file 8179799.f1.zip › Supplementary Figure1 (1).pdf]
